# Reply to Izuma and Kakinuma: Conversation aligns self-views above and beyond noise reduction

**DOI:** 10.1073/pnas.2508437122

**Published:** 2025-06-20

**Authors:** Christopher Welker, Thalia Wheatley, Meghan Meyer

**Affiliations:** ^a^Department of Psychological and Brain Sciences, Dartmouth College, Hanover, NH 03755; ^b^Santa Fe Institute, Santa Fe, NM 87501; ^c^Department of Psychology, Columbia University, New York, NY 10027

Izuma and Kakinuma demonstrate that repeated sampling reduces variability in self-reports, which can create the appearance of convergence over time (1). They raise an important point, and we are grateful to address it here. Our data show that the conversation-induced alignment we observed—what we called inter-self alignment (ISA; 2)—persists above and beyond this artifact. We compared our conversation condition to the data reported by Izuma and Kakinuma and a new control group that paralleled our original design, but without conversation. In both cases, conversation produced significantly greater alignment, indicating that the ISA effect cannot be explained by noise reduction alone. Data and analysis code are available at https://osf.io/8x5g2/.

**Fig. 1.**
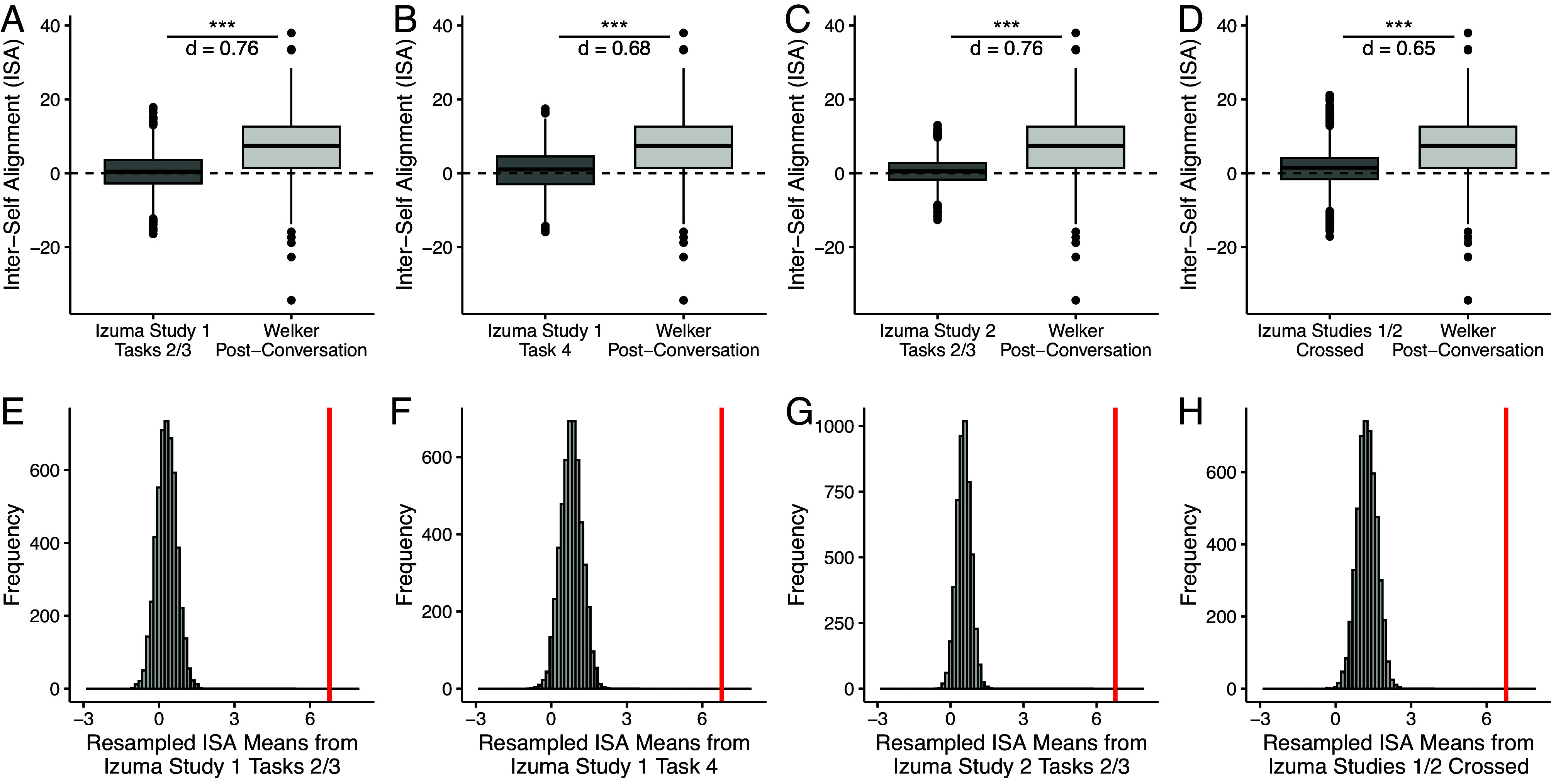
Panels (*A*–*D*) Boxplots showing differences in ISA between Izuma and Kakinuma’s studies and the original conversation study. The comparisons mirror those made by Izuma and Kakinuma in their figure 2 (i.e., direct comparison of means; 1). Conversation-related ISA was consistently greater than the alignment from repeated measurements documented by Izuma and Kakinuma. *** indicates *P* < 0.001 and t’s > 5.97. Panels (*E*–*H*) Histograms of 5,000 resampled means from Izuma and Kakinuma’s studies. Each resampling took the mean of 144 randomly selected ISA values from Izuma and Kakinuma’s data without replacement. The vertical red bar represents the mean of the postconversation data in the original study. In all comparisons, the conversation mean falls above all sample means from the control distribution.

First, we compared our conversation-induced ISA to the values reported by Izuma and Kakinuma. As shown in Fig. 1 *A*–*D*, ISA following conversation was significantly greater than ISA from repeated measures without conversation. To account for differences in sample size, we next generated resampled distributions of 5,000 sample means from Izuma and Kakinuma’s nonconversation ISA data (N = 144 per resample, selected randomly without replacement). In every case, the mean conversation-induced ISA exceeded the maximum values in the resampled distributions (Fig. 1 *E*–*H*).

**Fig. 2.**
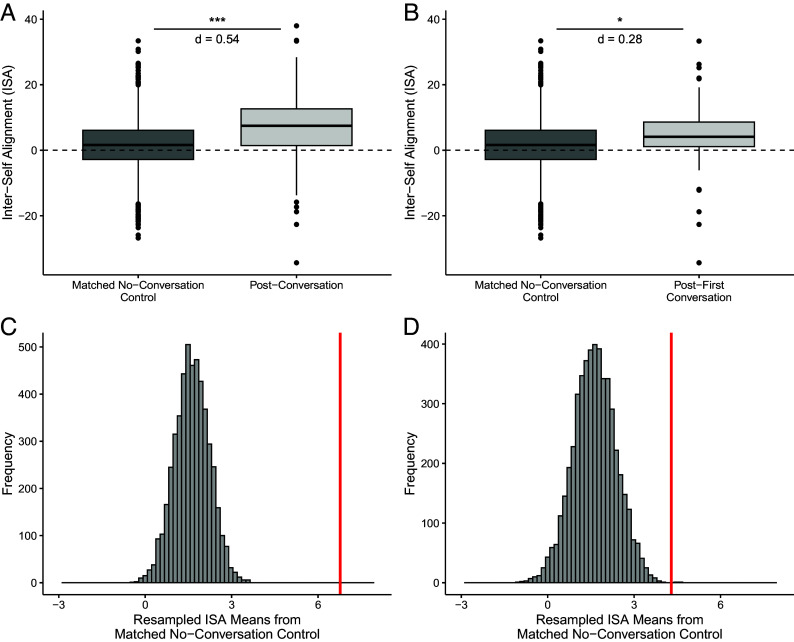
Panel (*A*) Boxplots showing the significant difference in ISA between the matched, no-conversation control group and the original study conversation group, *t*(152.61) = 5.49, 95% CI = [3.30, 7.00], *P* < 0.001, d = 0.54. Panel (*B*) Boxplots showing the significant difference in ISA between the matched, no-conversation control and baseline-to-first conversation in the original study, *t*(99.25) = 2.34, 95% CI = [0.41, 4.94], *P* = 0.021, d = 0.28. Panel (*C*) Histogram showing 5,000 resampled means (n = 144, without replacement) from the matched no-conversation control. The red bar marks the original study’s postconversation ISA mean, which exceeds all control samples (*P* < 0.001). Panel (*D*) Histogram showing 5,000 resampled means from the matched, no-conversation control. Because 96 data points comprised the baseline-to-first conversation mean (one per participant), each resample took the mean of 96 randomly selected ISA values without replacement. The red bar marks the original study’s baseline-to-first conversation ISA mean, which exceeds the control distribution (*P* < 0.001).

Second, we collected a new control group that more precisely matched the timing, structure, and sample demographics (M_age_ = 20.42, N_male_ = 33, N_female_ = 25, N_other_ = 6) of the original conversation condition. Participants provided informed consent (Columbia University IRB protocol AAAU7311), completed self-view trait ratings and, 24-h later, were contacted to fill out the ratings again. The 24-h minimum gap in time mirrored the gap between conversation partners’ baseline and first-conversation responses in the original study. As in the original study, trait order was randomized in each session. Both group mean comparisons and resampled distribution comparisons showed that the mean ISA in the conversation condition—alignment across all conversations and specifically from baseline to postfirst conversation responses—significantly exceeded alignment in the matched control group (Fig. 2).

Moreover, participants in the matched, no-conversation control group did not move significantly closer to the normative self-view (*t*(63) = 1.11, 95% CI = [−0.47, 1.63], *P* = 0.273, d = 0.14), unlike participants after a single conversation (*t*(95) = 3.40, 95% CI = [0.98, 3.73], *P* = 0.001, d = 0.35). A direct comparison confirmed the difference between groups (*t*(157.31) = 2.03, 95% CI = [0.05, 3.49], *P* = 0.043, d = 0.31). The conversation effect was also significantly greater than a bootstrapped distribution of the matched control group sample means (*P* < 0.001, N = 96 per resample, data selected randomly with replacement).

In sum, comparing our data to three independent no-conversation datasets, we find that conversation-induced ISA exceeds what would be expected from repeated sampling alone. Our analyses do not diminish the value of Izuma and Kakinuma’s response. Indeed, we replicate their findings; our control group demonstrated significant alignment without any conversation (*P* < 0.001). Nevertheless, the self-view alignment from conversation exceeds what can be attributed to responding to the same scale multiple times.
